# Mn^3+^/Mn^4+^ ion-doped carbon dots as fenton-like catalysts for fluorescence dual-signal detection of dopamine

**DOI:** 10.3389/fbioe.2022.964814

**Published:** 2022-09-07

**Authors:** Peide Zhu, Xuelin Zhao, Yuqi Zhang, Yinping Liu, Ziyi Zhao, Ziji Yang, Xinzhu Liu, Weiye Zhang, Zixuan Guo, Xiao Wang, Yingchun Niu, Meng Xu

**Affiliations:** ^1^ State Key Laboratory of Heavy Oil Processing, China University of Petroleum-Beijing, Beijing, China; ^2^ Department of Musculoskeletal Tumor, Senior Department of Orthopedics, Fourth Medical Center of PLA General Hospital, Beijing, China; ^3^ Medical School of Chinese PLA, Beijing, China; ^4^ Senior Department of Burns and Plastic Surgery, Fourth Medical Center of PLA General Hospital, Beijing, China

**Keywords:** Mn^3+^ /Mn^4+^ ion-doped CDs, fenton-like catalysis, dopamine, fluorescent probe, detection

## Abstract

Carbon dots (CDs), a new zero-dimensional material, have ignited a revolution in the fields of sensing, bioimaging, and biomedicine. However, the difficulty of preparing CDs with Fenton-like catalytic properties has seriously hindered their application in the diagnosis of oxidation/reduction biomolecules or metal ions. Here, an innovative method was successfully established to synthesize Mn^3+^/Mn^4+^ ion-doped blue-green fluorescent CDs with Fenton-like catalytic properties using manganese acetate as the manganese source. Specifically, the CDs prepared here were equipped with functional groups of -COOH, NH_2_, C=O, and Mn-O, offering the possibility to function as a fluorescence sensor. More importantly, the introduction of manganese acetate resulted in the preparation of CDs with Fenton-like catalytic properties, and the dual-signal fluorescence detection of dopamine (DA) was realized with linear ranges of 100–275 nM and 325–525 nM, and the detection limits were 3 and 12 nM, respectively. In addition, due to the Fenton-like catalytic activity of Mn^3+^/Mn^4+^ ion-doped CDs, the material has broad application prospects in the detection of oxidation/reduction biomolecules or metal ions related to disease diagnosis and prevention.

## Introduction

Up to now, the research on brain science has become more and more attractive in elucidating the structure and function of the human brain, as well as human behavior and mental activity, which involves enhancing human neural activity and improving the level of prevention, diagnosis, and treatment services for nervous system diseases (Poo and Tan, 2016). Dopamine (DA), a neurotransmitter, plays a crucial role in mood, sleep, memory, endocrine regulation, and movement (He et al., 2020; Wang et al., 2021). The level of DA in the brain of healthy people ranges from 1.3 to 2.6 μmol, and abnormal DA levels often lead to various diseases, including anorexia, epilepsy, Alzheimer’s disease, schizophrenia, and attention deficit dysfunction (Chen and Zhou, 2015; He et al., 2018). In addition, DA has been widely used in cell interfaces, drug delivery, biosensing coatings, and antibacterial research due to its rich amino and catechin functional groups on its surface (Natan et al., 2019). Therefore, considering the importance of DA in neurotransmission, drug delivery, and disease treatment, it is crucial to determine the concentration of DA *in vitro*.

To date, many methods for detecting DA have been developed to speed up the diagnosis and prevention of DA-related diseases to some extent, such as high-performance liquid chromatography (HPLC), electrochemistry, etc (Ngo et al., 2017; Du et al., 2019; Zhao et al., 2021). However, these methods usually have the disadvantages of expensive instruments and equipment, professional technology, and long time-consuming, which limit the broad application of the above-mentioned DA detection (Zhao et al., 2021). In contrast, the fluorescence detection method has the advantages of high sensitivity, convenient instruments, and low cost, which overcomes the methods above-mentioned for detecting DA (Du et al., 2019; Wang J. G et al., 2019). In recent years, researchers have developed a variety of fluorescent materials, including AIE dyes, upconversion nanoparticles, fluorescent metal nanoclusters, carbon dots (CDs), and other fluorescent materials (Wang et al., 2010; Tu and Wang, 2013; Yang et al., 2015; Anjali Devi et al., 2017; Luo et al., 2019). Therein, CDs are widely used in biosensing and bioimaging due to their excellent photostability and biocompatibility (Xu et al., 2021; Liu et al., 2013; He et al., 2018). For example, Xu et al. constructed a fluorescence resonance energy transfer (FRET) detection platform based on the synthesized yellow fluorescent CDs. This platform had high selectivity and sensitivity, and could specifically detect l-threonine in samples with the detection range of 0.1–0.5 mM (Xu et al., 2021). Based on the FRET effect, Liang et al. prepared a novel hybrid proportional fluorescence probe of CDs-gold nanoclusters that could detect DA in the 5–180 nM range (He et al., 2018). Zhou et al. synthesized long-wavelength emission fluorescent CDs by the one-pot hydrothermal method. The prepared CDs showed bright red fluorescence in different states, which could be used for diagnostic imaging of precious metal ions *in vivo* and *in vitro* (Gao et al., 2018). However, to the best of our investigation literature, the specific recognition of DA by Mn^3+^/Mn^4+^ ion-doped CDs as a dual-signal fluorescence sensing platform has not been reported.

**SCHEME 1:**
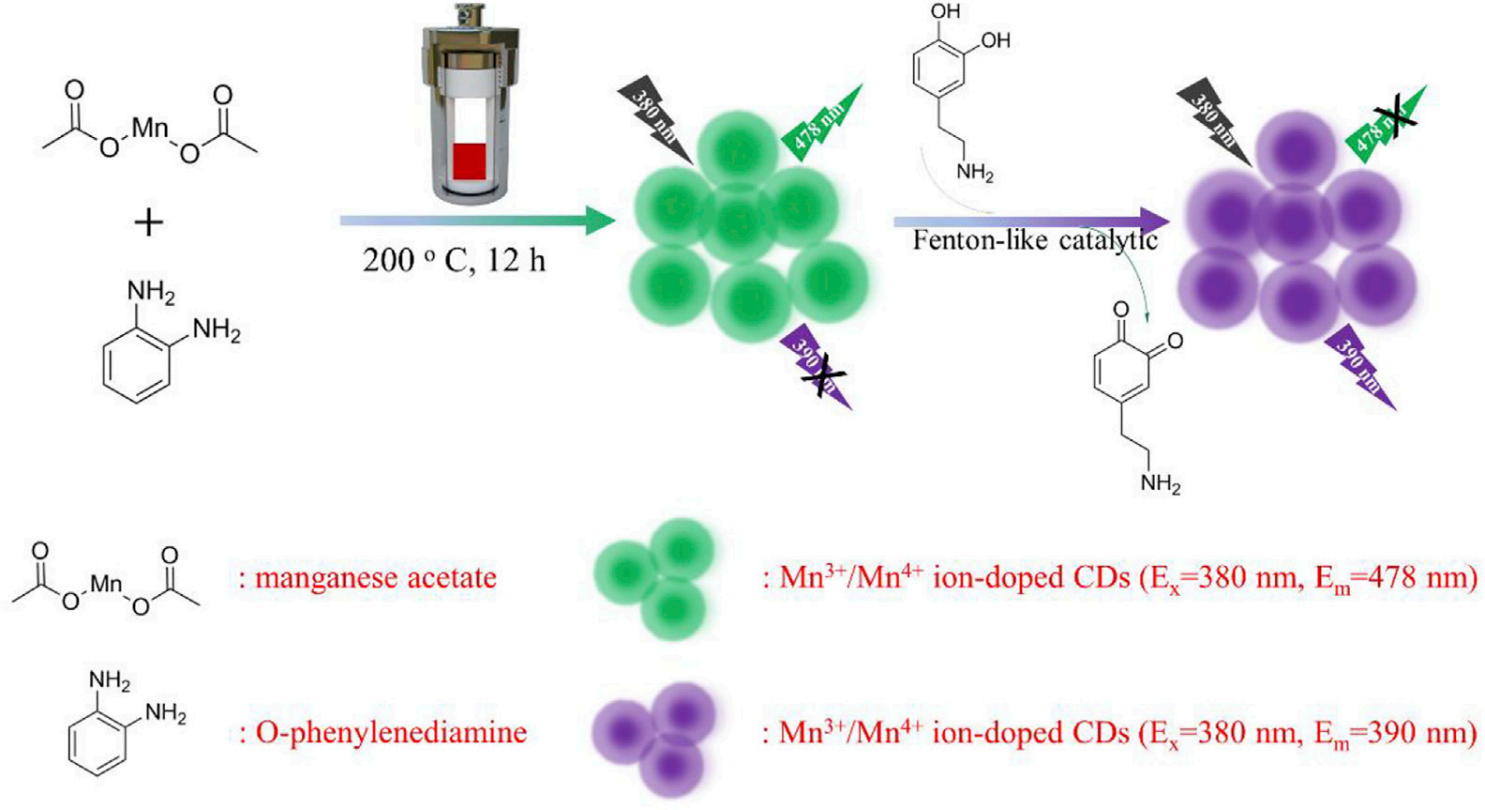
Schematic diagram of Mn^3+^/Mn^4+^ ion-doped CDs preparation and its application in DA detection.

As shown in Scheme 1, manganese acetate and O-phenylenediamine were used as manganese and carbon sources to design synthesis Mn^3+^/Mn^4+^ ion-doped CDs by hydrothermal method. DA is an important organic chemical in the catechol family that plays an important role in human motor, neuroendocrine regulation, disease diagnosis, and drug delivery. However, clinical DA is often replaced with DA hydrochloride and hydrogen bromide due to its susceptibility to oxidation in the air and light. Mn^3+^/Mn^4+^ ion-doped CDs had good optical stability and Fenton-like catalytic properties; and could detect DA with dual-response fluorescence with linear ranges of 100–275 nM and 325–525 nM, and detection limits of 3 and 12 nM, respectively. Therefore, because of its wide linear range and low detection limit, it has a broad application prospect in diagnosing and preventing DA-related diseases. In addition, the material has a good role and effect in the detection of oxidation/reduction biomolecules or metal ions, and can be widely used in the detection and diagnosis caused by abnormalities of related biomolecules or metal ions.

## Experimental section

### Materials

O-phenylenediamine, ascorbic acid (AA), glycine (Gly), glutathione (GSH), dopamine (DA), iron chloride, bismuth chloride, hafnium chloride, hydrochloric acid, indium chloride, and lysine (Lys) were obtained from Aladdin Reagent Co., Ltd. Sulfamic acid (SA), manganese acetate, NaCl, copper chloride, manganese chloride, N, N-dimethylformamide (DMF), and NaOH were gained from Tianjin Guangfu Fine Chemicals Co., Ltd. The ultrapure water in the whole experiment was prepared by the BK-10B system.

### Characterization

The particle size of Mn^3+^/Mn^4+^ ion-doped CDs was recorded by a JEM-2100 transmission electron microscope (TEM). The height distribution of Mn^3+^/Mn^4+^ ion-doped CD was detected by atomic force microscopy (AFM). The UV-vis absorption and fluorescence spectra of Mn^3+^/Mn^4+^ ion-doped CDs were analyzed by an FS5 spectrophotometer. Fourier transform infrared (FT-IR) spectra of Mn^3+^/Mn^4+^ ion-doped CDs were measured by Bruker spectrometer. The structure of Mn^3+^/Mn^4+^ ion-doped CDs was recorded by X-ray diffraction (XRD) using Cu Kα radiation at a voltage of 40 kV and current of 40 mA. X-ray photoelectron spectroscopy (XPS) of Mn^3+^/Mn^4+^ ion-doped CDs was obtained by ESCALAB 250 Xi electron spectrometer.

### Synthesis of Mn^3+^/Mn^4+^ ion-doped CDs

During the stirring process, 0.31 g O-phenylenediamine and 0.331 g manganese acetate were dissolved in 8 ml DMF solution (containing 1 ml (1%) acetic acid) to form a mixed solution. The above-mixed solution was transferred to a 25 ml reaction vessel and heated at 200°C for 12 h. Subsequently, the solution was centrifuged to remove the solid precipitates at room temperature. Finally, solid Mn^3+^/Mn^4+^ ion-doped CDs were obtained by rotation.

### Detection of DA using Mn^3+^/Mn^4+^ ion-doped CDs

Add different contents of DA (0–0.55 μM) aqueous solution to 3 ml of Mn^3+^/Mn^4+^ ion-doped CDs. The fluorescence spectra were then measured at 380 nm excitation. Selectivity was achieved by adding other biomolecules (including SA, Gly, GSH, AA, Bi^3+^, Cu^2+^, Hf^4+^, Fe^3+^, In^3+^, Mn^2+^, Na^+^, and Lys) instead of DA. The competitive experiments were carried out by adding DA to the Mn^3+^/Mn^4+^ ion-doped CDs solution.

## Results and discussion

### Characterization of Mn^3+^/Mn^4+^ ion-doped CDs

**FIGURE 1:**
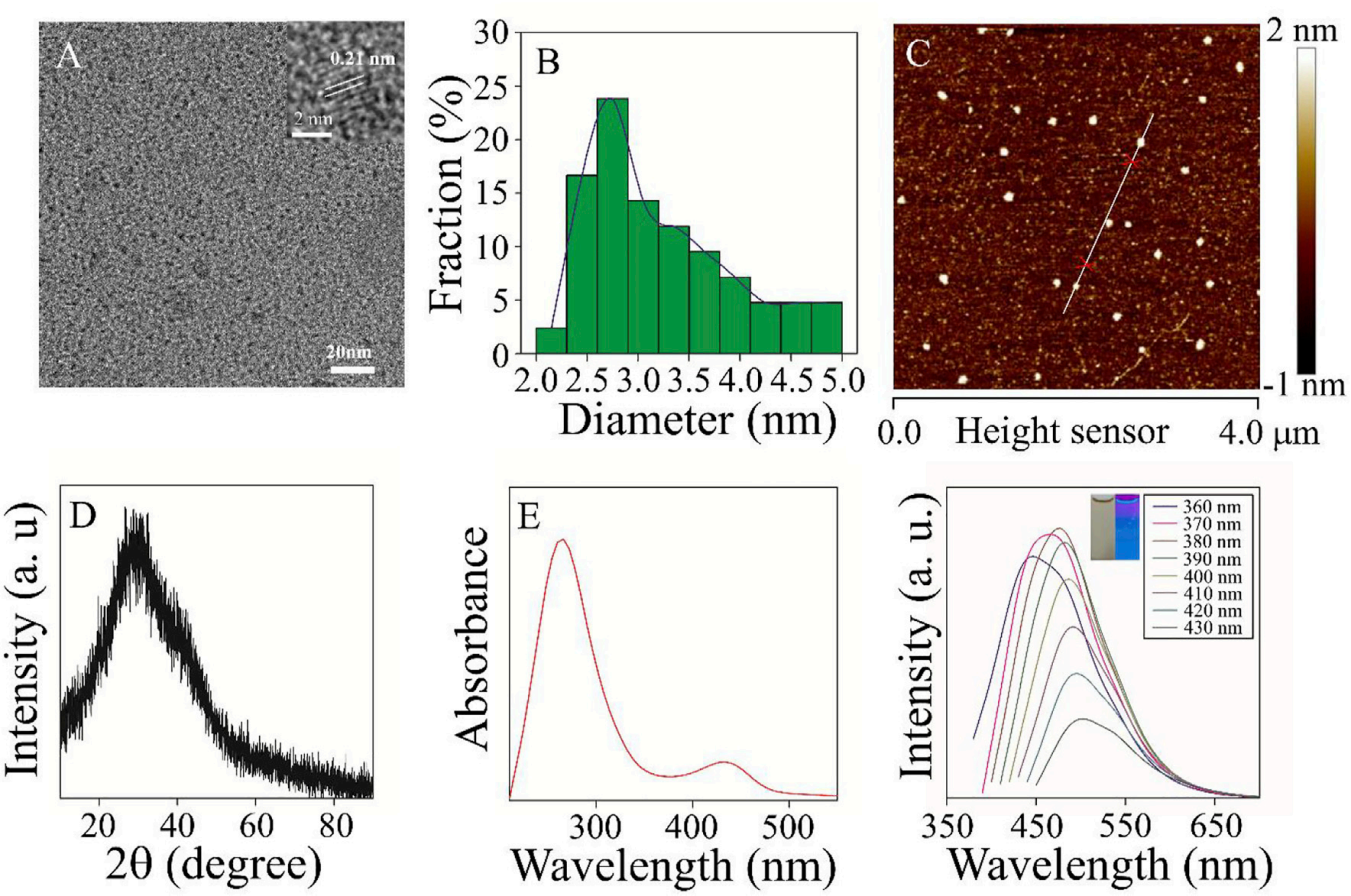
The morphology and optical properties of the samples were characterized **(A)** TEM and HRTEM (inset) images of Mn^3+^/Mn^4+^ ion-doped CDs **(B)** TEM particle size distribution. **(C)** AFM image of Mn^3+^/Mn^4+^ ion-doped CDs **(D)** XRD image of Mn^3+^/Mn^4+^ ion-doped CDs. **(E)** UV-vis absorption spectra of Mn^3+^/Mn^4+^ ion-doped CDs. (F) The fluorescence emission spectra of Mn^3+^/Mn^4+^ ion-doped CDs at different excitation wavelengths of 340–440 nm.

The structure, morphology, and optical properties of Mn^3+^/Mn^4+^ ion-doped CDs were studied under heating at 200°C for 12 h. The TEM image of Mn^3+^/Mn^4+^ ion-doped CDs was shown in Figures 1A,B, and the as-prepared CDs exhibited good dispersion and uniform size of quasi-spherical shape with an average particle size of 3.5 nm (Figure 1B). Furthermore, the HRTEM in the inset of Figure 1A showed that the lattice spacing of the Mn^3+^/Mn^4+^ ion-doped CDs was 0.21 nm, corresponding to the (002) crystal plane of graphite carbon (Tang et al., 2018). In addition, as shown in Figure 1C, Supplementary Figures S1, S2, the height and morphology of Mn^3+^/Mn^4+^ ion-doped CDs were further studied by AFM. The AFM image showed that the height of Mn^3+^/Mn^4+^ ion-doped CDs was about 2.0 nm (Supplementary Figures S1, S2), similar to the TEM results. The X-ray diffraction (XRD) pattern of Mn^3+^/Mn^4+^ ion-doped CDs was shown in Figure 1D. A new wide diffraction peak appears in the XRD pattern of the CDs, located at 26.8°C, which is related to the (002) crystal plane of the graphitic carbon material (Tian et al., 2017; Gao et al., 2018). The aqueous solution of Mn^3+^/Mn^4+^ ion-doped CDs showed two UV-vis absorption peaks at 270 and 434 nm, as shown in Figure 1E. The absorption peak at 270 nm was attributed to the π-π conjugation of the benzene ring, which exhibited a lower energy absorption band at approximately 434 nm (Shi et al., 2020). The fluorescence emission spectra of Mn^3+^/Mn^4+^ ion-doped CDs are shown in Figure 1F. It can be seen that the fluorescence emission peak moved in the direction of a long wavelength, and the fluorescence intensity changed with the increase of the excitation wavelength. The illustration in Figure 1F shows the fluorescent color of Mn^3+^/Mn^4+^ ion-doped CDs. These fluorescence characteristics may be related to the functional groups on the surface of Mn^3+^/Mn^4+^ ion-doped CDs, and various functional groups can introduce different defects into the surface of Mn^3+^/Mn^4+^ ion-doped CDs as excitation energy traps, resulting in different fluorescence properties (Zhao et al., 2018). In addition, photostability is the premise of Mn^3+^/Mn^4+^ ion-doped CDs for biomolecular or metal ion detection and imaging. We studied the changes in fluorescence intensity of Mn^3+^/Mn^4+^ ion-doped CDs under different salt solution concentrations, time, and pH values. As shown in Supplementary Figures S3, S4, the fluorescence intensity of Mn^3+^/Mn^4+^ ion-doped CDs remain almost unchanged with the increase in salt solution concentration and time. Based on this, we chose to carry out the related experiments of DA detection in the later stage without sodium chloride solution. As shown in Supplementary Figure S5, the fluorescence intensity of Mn^3+^/Mn^4+^ ion-doped CDs is relatively low under strong acid or alkali conditions. Therefore, we choose pH 7 as the best condition for the anti-interference experiment of DA and related metal ions or biomolecules. Under the optimum conditions, the highest quantum yield of Mn^3+^/Mn^4+^ ion-doped CDs is 4.29%.

**FIGURE 2:**
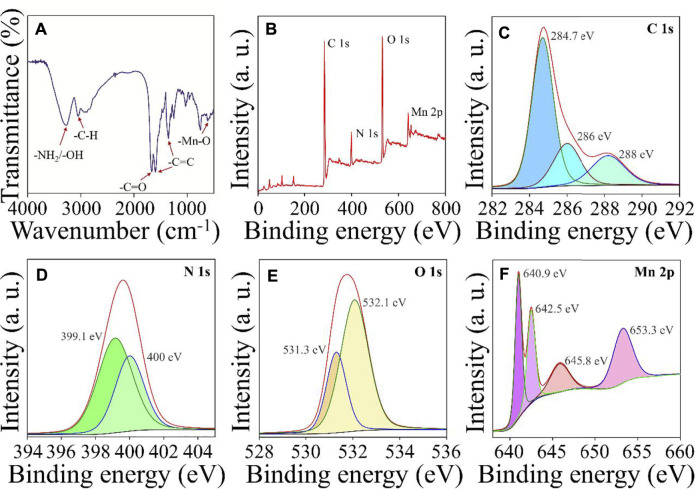
Structural characterization of Mn^3+^/Mn^4+^ ion-doped CDs **(A)** FT-IR spectra of Mn^3+^/Mn^4+^ ion-doped CDs. **(B)** the total XPS spectra of Mn^3+^/Mn^4+^ ion-doped CDs and high-resolution C 1s **(C)**, N 1s **(D)**, O 1s **(E)**, and Mn 2p **(F)** spectra of Mn^3+^/Mn^4+^ ion-doped CDs.

FT-IR and XPS evaluated the chemical composition and functional groups of Mn^3+^/Mn^4+^ ion-doped CDs. FT-IR spectra were shown in Figure 2A, the peak of Mn^3+^/Mn^4+^ ion-doped CDs were 3,367.8, 1,680.9, 1,600, 1,400, and 610 cm^−1^, respectively, which were attributed to -NH_2_/-OH, C=O, benzene ring, and Mn-O functional groups (Zhu et al., 2020). These results indicated abundant functional groups on the surface of Mn^3+^/Mn^4+^ ion-doped CDs, which makes Mn^3+^/Mn^4+^ ion-doped CDs have better dispersibility in an aqueous solution. In addition, XPS also further confirmed the presence of C=O, C=C, -NH_2_/-OH and Mn-O chemical structures and elemental compositions on the surface of Mn^3+^/Mn^4+^ ion-doped CDs. The total spectrum of XPS shown in Figure 2B was mainly composed of C 1s, N 1s, O 1s, and Mn 2p, with the proportion of elements being 69.96, 8.55, 19.13, and 2.37% (Supplementary Table S1), respectively. As shown in Figure 2C, the high-resolution spectrum of C 1s showed three prominent peaks, and the binding energies of 284.7, 286, and 288 eV correspond to C=C, C-N, and C=O functional groups, respectively. The high-resolution spectrum of O 1s (Figure 2D) could be decomposed into two binding energies of 531.3 and 532.1 eV, which could be attributed to Mn-O and C-O, respectively. There were two binding energies of 399.1 and 400 eV in the high-resolution spectrum of N 1s, which could be attributed to C-N and N-H functional groups (Figure 2E). Moreover, Figure 2F shows the XPS spectrum of Mn 2p in the Mn^3+^/Mn^4+^ ion-doped CDs with four prominent peaks at 640.9, 645.8 eV, and 642.5, 653.3 eV, respectively, indicating that Mn^3+^ and Mn^4+^ ions exist in the surface of Mn^3+^/Mn^4+^ ion-doped CDs, and the ratio of Mn^3+^/Mn^4+^ on the surface was 0.82 (Wang J et al., 2019). FT-IR and XPS spectra showed a large number of hydrophilic functional groups on the surface of Mn^3+^/Mn^4+^ ion-doped CDs, which not only provided interaction sites for specific ions or biomolecules; but also improved the biocompatibility of the material itself.

### Fluorescence detection of DA

**FIGURE 3:**
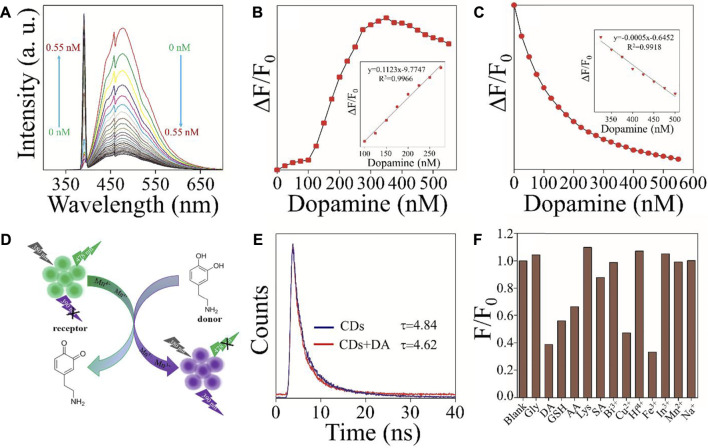
Mn^3+^/Mn^4+^ ion-doped CDs for DA detection **(A)** Effect of DA content on the fluorescence intensity of Mn^3+^/Mn^4+^ ion-doped CDs. **(B)** The curve relationship between fluorescence intensity at 390 nm and DA (0–550 nM) content (inset: the linear relationship between ΔF/F_0_ and DA content (100–275 nM)) **(C)** The curve relationship between fluorescence intensity at 478 nm and DA (0–550 nM) content (inset: the linear relationship between ΔF/F_0_ and DA (325–525 nM) content **(D)** Schematic illustration of the mechanism of Mn^3+^/Mn^4+^ ion-doped CDs for DA detection. **(E)** The fluorescence decay curves of Mn^3+^/Mn^4+^ ion-doped CDs in an aqueous solution without and with DA. **(F)** Anti-interference of fluorescence intensity of Mn^3+^/Mn^4+^ ion-doped CDs at 478 nm.

Due to Mn^3+^ and Mn^4+^ions on the surface of CDs, the material had Fenton-like catalytic properties. The effect of DA on the fluorescence intensity of Mn^3+^/Mn^4+^ ion-doped CDs at 390 and 478 nm was studied under optimal conditions. As shown in Figure 3A, with the gradual increase of DA content, the fluorescence intensity of Mn^3+^/Mn^4+^ ion-doped CDs at 478 nm showed a continuous decrease, and the fluorescence intensity at 390 nm showed a continuously increasing trend. Figures 3B,C illustrate the linear relationship between ΔF/F_0_ (ΔF = F-F_0_, where F_0_ and F are the fluorescence intensities of Mn^3+^/Mn^4+^ ion-doped CDs in the absence and presence of DA, respectively) and DA content. The linear range of Figure 3C was 100–275 nM with a correlation coefficient of 0.9966, and the detection limit was as low as 3 nM (n = 3); the linear range of Figure 3D was 325–525 nM with a correlation coefficient of 0.9918, and the detection limit was as low as 12 nM (n = 3). This value is equivalent to or even better than the previous fluorescence detection of DA (Table 1), indicating that this platform is suitable for the determination of DA.

**TABLE 1:** The comparison of this method with other DA detection in literature.

Fluorescence sensor	Linear range	LOD	Ref.
N, P-CQDs	10–500 μM	0.021 mM	Yang et al. (2015)
GQDs	0.25–50 μM	0.09 mM	Zhao et al. (2016)
QDs@silica	0.5–100 μM	0.24 mM	(Qiang et al., 2012)
CDs	25–500 μM	0.7 nM	(Niu et al., 2012)
CuInS_2_ QDs	0.5–40 μM	200 nM	(Su et al., 2013)
Mn^3+^/Mn^4+^ ion-doped CDs	100–275 nM	3 nM	This work
	325–525 nM	12 nM	

To study the mechanism of Mn^3+^/Mn^4+^ ion-doped CDs as a fluorescent probe for detecting DA, the valence states of Mn ion in Mn^3+^/Mn^4+^ ion-doped CDs without and containing DA were analyzed, respectively. As shown in Figure 2F and Supplementary Figure S6, compared with Mn^3+^/Mn^4+^ ion-doped CDs without DA, the Mn^3+^/Mn^4+^ ratio of DA-containing Mn^3+^/Mn^4+^ ion-doped CDs increased from 0.82 to 1.04. These results suggest that the mechanism of Mn^3+^/Mn^4+^ ion-doped CDs and DA was a redox property. As shown in Figure 3D, when DA was added to the aqueous solution containing Mn^3+^/Mn^4+^ ion-doped CDs, the DA reacted with Mn^4+^ ion on the surface of CDs, resulting in the conversion of phenolic hydroxyl functional groups on DA into O-quinone structure (Shi et al., 2020). At the same time, Mn^4+^ ions on the surface of CDs act as oxidants to obtain electrons, which changes the Mn^3+^/Mn^4+^ ratio on the surface of CDs, resulting in an increase or decrease in the fluorescence intensity of emission peak at 390 and 478 nm of Mn^3+^/Mn^4+^ ion-doped CDs with the rise of DA content. In addition, we also carried out the time-dependent single-photon counting spectra of Mn^3+^/Mn^4+^ ion-doped CDs under different conditions. As shown in Figure 3E, the average fluorescence lifetime of single Mn^3+^/Mn^4+^ ion-doped CDs was 4.82 ns. The average fluorescence lifetime of Mn^3+^/Mn^4+^ ion-doped CDs decreased to 4.62 ns after adding DA. The decreased average fluorescence lifetime indicates that the electron transfer between Mn^3+^/Mn^4+^ ion-doped CDs and DA was a dynamic fluorescence quenching induced process. Between them, Mn^3+^/Mn^4+^ ion-doped CDs and DA were used as energy receptors and energy donors, respectively. To induce Fenton-like catalysis between catechol functional groups on DA molecule and Mn^3+^/Mn^4+^ ion pairs on CDs surface, jointly constructing donor/receptor pairs of FRET.

Mn^3+^/Mn^4+^ ion pairs with Fenton-like catalytic properties exist on the surface of CDs, which have the potential to be used as fluorescent probes for oxidation/reduction of biomolecules or metal ions. DA, a suitable electron donor, will quench and enhance the fluorescence intensity at 478 and 390 nm respectively after mixing with Mn^3+^/Mn^4+^ ion-doped CDs. Firstly, we studied the fluorescence intensity changes of Mn^3+^/Mn^4+^ ion-doped CDs in NaCl solutions with different concentrations. It can be seen that the fluorescence intensity of Mn^3+^/Mn^4+^ ion-doped CDs was not affected by the NaCl solution. Secondly, the effects of biomolecules or metal ions on the fluorescence intensity of Mn^3+^/Mn^4+^ ion-doped CDs were also investigated. As shown in Figure 3F and Supplementary Figure S7, biomolecules or metal ions such as Lys, Gly, Bi^3+^, Hf^4+^, In^3+^, Mn^2+^, and Na^+^ had almost no influence on the fluorescence intensity ratio F/F_0_ of Mn^3+^/Mn^4+^ ion-doped CDs. SA had a weak fluorescence quenching effect on the fluorescence intensity ratio F/F_0_ of Mn^3+^/Mn^4+^ ion-doped CDs, indicating that Mn^3+^/Mn^4+^ ion-doped CDs had relatively good selectivity for the above biomolecules or metal ions. Finally, to verify the Fenton-like catalytic properties of Mn^3+^/Mn^4+^ ion-doped CDs, we further studied the effect of oxidation/reduction biomolecules or metal ions on the fluorescence intensity ratio F/F_0_ of Mn^3+^/Mn^4+^ ion-doped CDs. As shown in Figure 3F and Supplementary Figure S7, after adding oxidation/reduction biomolecules or metal ions such as AA, GSH, Cu^2+^, and Fe^3+^, the fluorescence intensity ratio F/F_0_ of Mn^3+^/Mn^4+^ ion-doped CDs at 478 nm was relatively lower than that of the blank control group. At the same time, the fluorescence intensity ratio F/F_0_ of Mn^3+^/Mn^4+^ ion-doped CDs at 390 nm was higher than that of the blank control group. The results indicate that Mn^3+^/Mn^4+^ ion-doped CDs have the potential as fluorescence probes for the detection of oxidation/reduction biomolecules or metal ions. However, the interference of other oxidation/reduction biomolecules or metal ions should be avoided during the detection of a certain oxidation/reduction biomolecule or metal ions, to enhance the accuracy and reliability of Mn^3+^/Mn^4+^ ion-doped CDs.

## Conclusion

In conclusion, we reported a simple hydrothermal method for the synthesis of Mn^3+^/Mn^4+^ ion-doped blue-green fluorescent CDs. These CDs showed excellent photostability and Fenton-like catalytic properties; and could be used as biosensors for DA detection with detection limits of 3 and 12 nM, respectively. In addition, due to the Fenton-like catalytic properties of CDs, Mn^3+^/Mn^4+^ ion-doped CDs have a certain fluorescence response to oxidation/reduction biomolecules or metal ions. Therefore, during the detection of oxidation/reduction biomolecules or metal ions by Mn^3+^/Mn^4+^ ion-doped CDs, the interference of other oxidation/reduction substances should be avoided so as to enhance the accuracy of fluorescent probes. The present work not only provides a new method for the synthesis of materials with Fenton-like catalysis; but also demonstrates the great potential of these materials for the detection of oxidation/reduction biomolecules or metal ions.

## Data Availability

The original contributions presented in the study are included in the article/Supplementary Material, further inquiries can be directed to the corresponding authors.
