# Supplementary material for: Mn3+/Mn4+ ion-doped carbon dots as fenton-like catalysts for fluorescence dual-signal detection of dopamine
Source: Front Bioeng Biotechnol. 2022 Sep 7;10:964814. doi: 10.3389/fbioe.2022.964814 (PMC9490222; doi:10.3389/fbioe.2022.964814)
Supplement: Supplementary file 1 [file Presentation1.pdf]

Supplementary Material

Mn^{3+}/Mn^{4+} ion-doped Carbon Dots as Fenton-like Catalysts for Fluorescence Dual-signal Detection of Dopamine

Peide Zhu[1=], Xuelin Zhao[2, 4=], Yuqi Zhang[1=], Yinping Liu[1], Ziyi Zhao[2], Ziji Yang[1], Xinzhu Liu[3], Weiye Zhang[1], Zixuan Guo[2], Xiao Wang[4], Yingchun Niu[1]*, Meng Xu[2*]

[1]State Key Laboratory of Heavy Oil Processing, China University of Petroleum-Beijing, Beijing 102249, China

[2]Department of Musculoskeletal Tumor, Senior Department of Orthopedics, the Fourth Medical Center of PLA General Hospital, Beijing 100142, China

[3]Senior Department of Burns and Plastic Surgery, the Fourth Medical Center of PLA General Hospital, Beijing 100142, China

[4]Medical School of Chinese PLA, Beijing, 100853, China

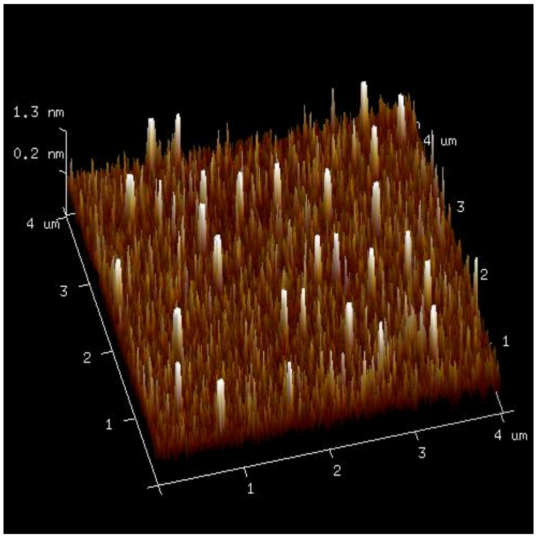

Fig. 1S. AFM 3D image of Mn^{3+}/Mn^{4+} ion-doped CDs.

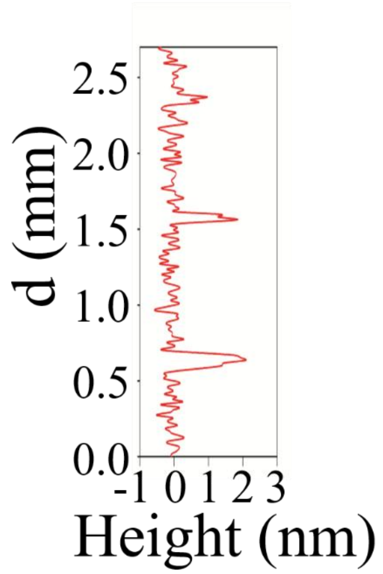

Fig. S2. The histogram of the height distribution of Mn^{3+}/Mn^{4+} ion-doped CDs was white in the 1C image.

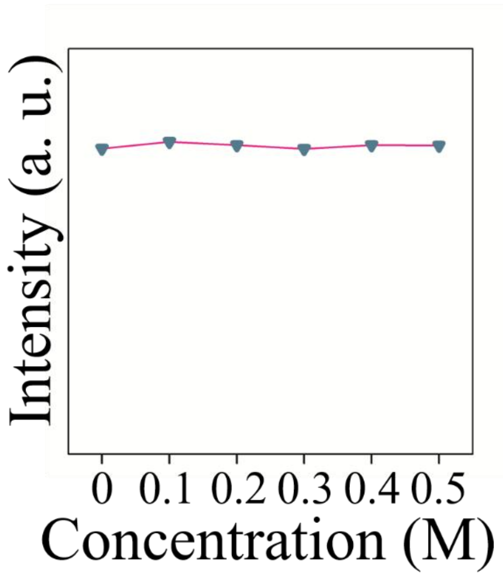

Fig. S3. Fluorescence spectra of Mn^{3+}/Mn^{4+} ion-doped CDs with different concentrations of NaCl solution at 380 nm excitation wavelength.

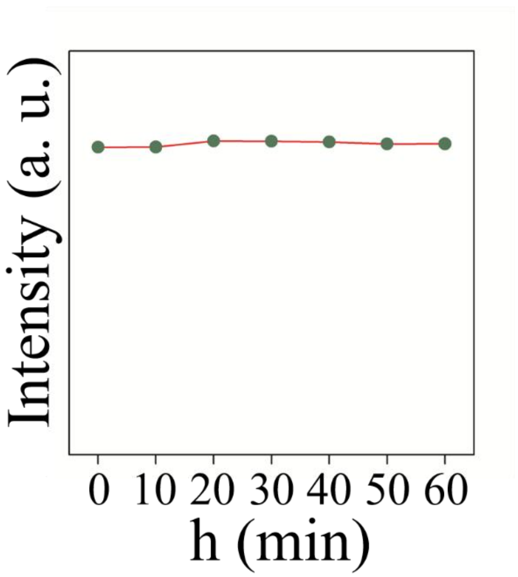

Fig. S4. Fluorescence spectra of Mn^{3+}/Mn^{4+} ion-doped CDs at 380 nm excitation wavelength at different times.

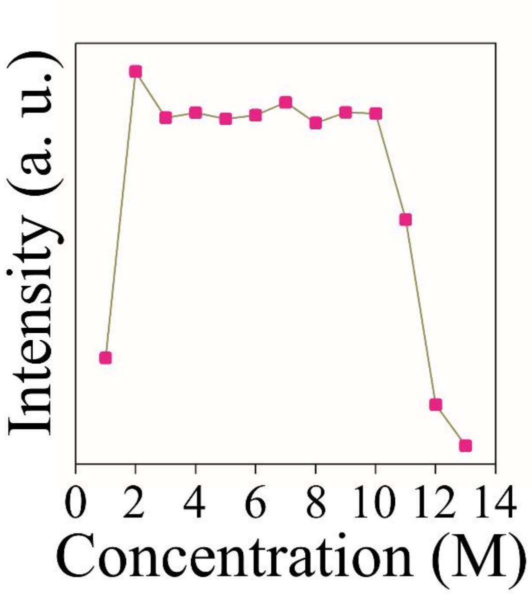

Fig. S5. Fluorescence spectra of Mn^{3+}/Mn^{4+} ion-doped CDs at 380 nm excitation wavelength at different pH values.

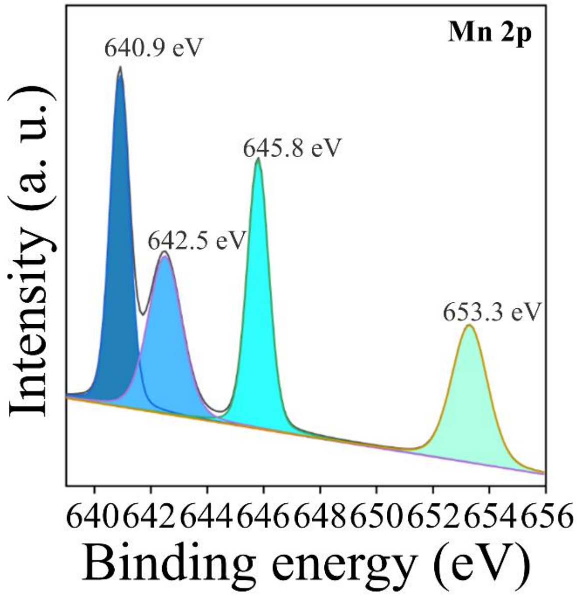

Fig. S6. High-resolution Mn 2p spectra of Mn^{3+}/Mn^{4+} ion-doped CDs with addition of DA.

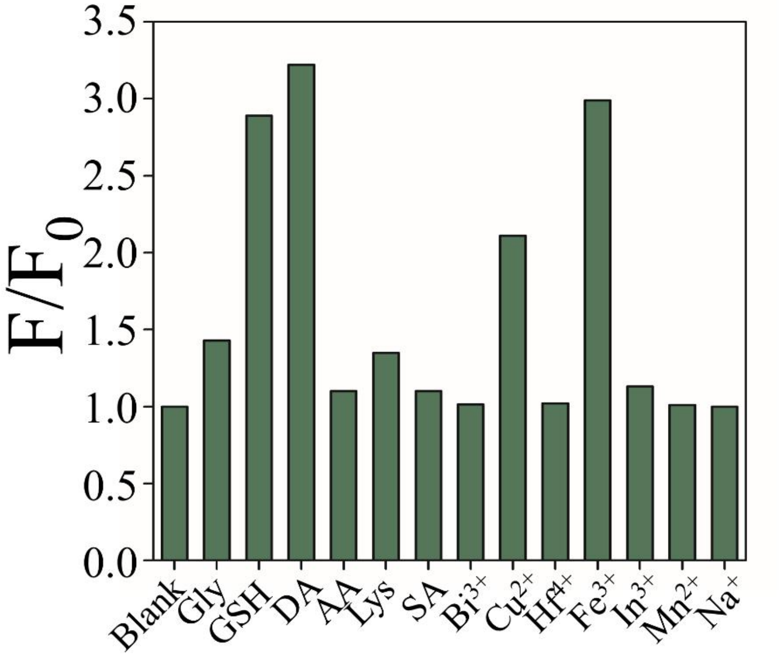

Fig. S6. Anti-interference of fluorescence intensity of Mn^{3+}/Mn^{4+} ion-doped CDs at 390 nm.

Table S1. The relative contents of C, N, O, and Mn atoms in the XPS spectra of Mn^{3+}/Mn^{4+} ion-doped CDs.

Mn^{3+}/Mn^{4+} ion-doped CDs.

C 1s (%)	N 1s (%)	O 1s (%)	Mn 2p (%)
69.96	8.55	19.13	2.37
